# Feasibility of ex vivo fluorescence imaging of angiogenesis in (non-) culprit human carotid atherosclerotic plaques using bevacizumab-800CW

**DOI:** 10.1038/s41598-021-82568-8

**Published:** 2021-02-03

**Authors:** Lydian A. Huisman, Pieter J. Steinkamp, Jan-Luuk Hillebrands, Clark J. Zeebregts, Matthijs D. Linssen, Annelies Jorritsma-Smit, Riemer H. J. A. Slart, Gooitzen M. van Dam, Hendrikus H. Boersma

**Affiliations:** 1 Department of Surgery, University of Groningen, University Medical Center Groningen, Groningen, The Netherlands; 2 Department of Clinical Pharmacy and Pharmacology, University of Groningen, University Medical Center Groningen, Hanzeplein 1, 9713 GZ Groningen, The Netherlands; 3 Department of Pathology and Medical Biology, Division of Pathology, University of Groningen, University Medical Center Groningen, Groningen, The Netherlands; 4 Department of Gastroenterology and Hepatology, University of Groningen, University Medical Center Groningen, Groningen, The Netherlands; 5 Medical Imaging Center, Department of Nuclear Medicine and Molecular Imaging, University of Groningen, University Medical Center Groningen, Groningen, The Netherlands; 6 Department of Biomedical Photonic Imaging, Faculty of Science and Technology, University of Twente, Enschede, The Netherlands; 7 Department of Surgery, Nuclear Medicine and Molecular Imaging and Intensive Care, University of Groningen, University Medical Center Groningen, Groningen, The Netherlands

**Keywords:** Biological techniques, Biomarkers, Cardiology, Medical research, Signs and symptoms

## Abstract

Vascular endothelial growth factor-A (VEGF-A) is assumed to play a crucial role in the development and rupture of vulnerable plaques in the atherosclerotic process. We used a VEGF-A targeted fluorescent antibody (bevacizumab-IRDye800CW [bevacizumab-800CW]) to image and visualize the distribution of VEGF-A in (non-)culprit carotid plaques ex vivo. Freshly endarterectomized human plaques (*n* = 15) were incubated in bevacizumab-800CW ex vivo. Subsequent NIRF imaging showed a more intense fluorescent signal in the culprit plaques (*n* = 11) than in the non-culprit plaques (*n* = 3). A plaque received from an asymptomatic patient showed pathologic features similar to the culprit plaques. Cross-correlation with VEGF-A immunohistochemistry showed co-localization of VEGF-A over-expression in 91% of the fluorescent culprit plaques, while no VEGF-A expression was found in the non-culprit plaques (*p* < 0.0001). VEGF-A expression was co-localized with CD34, a marker for angiogenesis (*p* < 0.001). Ex vivo near-infrared fluorescence (NIRF) imaging by incubation with bevacizumab-800CW shows promise for visualizing VEGF-A overexpression in culprit atherosclerotic plaques in vivo.

## Introduction

Carotid artery atherosclerosis is a leading cause for ischemic stroke, accounting for approximately one in four stroke cases^1^. The annual stroke risk due to carotid atherosclerosis is increased by 10–20% compared to patients without carotid artery stenosis^2^. A study based on the large population-based Rotterdam cohort study demonstrated that 80% of the cohort over 55 years of age had some degree of carotid artery atherosclerosis^3^. Risk factors for the development of atherosclerosis include hypertension, dyslipidemia, diabetes mellitus, obesity and smoking. Most of these risk factors are increasingly prevalent amongst the world population as the result of ageing and the earlier emergence of comorbidities^4^.

Formation of an atherosclerotic plaque within the vessel wall can cause partial or total vessel occlusion. Plaque rupture inevitably causes downstream ischemic damage in other organs^5^. Currently, there is no imaging modality available to stratify the risk of rupture in vivo. This leads to unnecessary surgical intervention in many patients, which is not desirable as the mortality rate is approximately 3% for this type of operation^6^. Identifying intraplaque biomarkers might possibly reveal the extent of plaque (in)stability and therefore aid in risk-stratification. Besides the composition and volume of the plaque, pathological plaque properties are crucial in assessing its stability. These properties include inflammation, lipid accumulation, angiogenesis, calcium precipitation, proteolysis, apoptosis, and thrombosis^5,7,8^*.* Ultimately, a combination of increased endothelial shear stress and increased tensile (wall) stress facilitates rupture of the unstable plaque^9^. Specific molecular imaging techniques can aid in predicting the risk of stroke by visualizing and quantifying plaque characteristics. Currently, carotid endarterectomy (CEA) is primarily performed on eligible patients with carotid stenotic lesions and signs of transient ischemic attacks or minor ischemic stroke to reduce the risk of future events. Imaging techniques might enable reliable selection for CEA surgery before symptom onset, in for example a high-risk patient population.

Positron emission tomography (PET) and single-photon emitted computed tomography (SPECT) have been investigated in detecting the vulnerable plaque. Although a recent meta-analysis found that ^18^F-FDG uptake was associated with inflammation in the symptomatic plaque, a clear cut-off point for the vulnerable plaque could not be established due to the heterogeneity of data previously described^10^. Another study reported that ^18^F-FDG uptake in recent transient ischemic attack (TIA) or amaurosis fugax patients did not differ significantly from asymptomatic patients, rendering it a questionable clinical indicator for plaque vulnerability^11^. ^18^F-sodium fluoride was investigated as a tracer for plaque calcification in both myocardial infarction and symptomatic carotid stenosis patients. The former observed uptake in almost all culprit plaques, but also in half of their non-culprit plaque group (stable angina pectoris patients)^12^. The latter did not observe a statistical difference between uptake of culprit and non-culprit carotid plaques, based on time interval between symptom onset and CEA^13^.

Ultrasound techniques have also been investigated in atherosclerotic plaques. Plaque surface irregularity was also investigated as risk indicator for cerebrovascular events. Kanber et al.^14^ demonstrated an accuracy of 83% in symptomatic patients with ultrasound imaging of plaque surface irregularities in combination with the degree of stenosis, whereas plaques from asymptomatic patients have a significantly smoother surface. However, it is unclear whether plaque surface irregularities were true plaque ulcerations or other, incidental, surface defects. Additionally, identification of irregularities depends on scanning resolution and might be missed due to two-dimensional ultrasound imaging.

A non-invasive clinical follow-up study was performed in patients who underwent carotid endartectomy, collecting test and imaging results from the hospitals concerned. During this study, a yearly risk assessment was performed based on analysis of the endarterectomized samples and the clinical patients characteristics, demonstrating that plaque characteristics such as macrophage infiltration and lipid core extent alone were not predictive of future cardiovascular events due to other atherosclerotic plaques^15^. A potential new target for plaque categorization is intraplaque angiogenesis^15–17^. Angiogenesis within the atherosclerotic plaque is considered to arise from initial inflammation of the arterial wall and subsequent influx of macrophages resulting in a relative localized hypoxia, the latter being the most important driver through expression of HIF-1α for vascular endothelial growth factor-A (VEGF-A) expression^18^,^19^. Consequently, intraplaque release of VEGF-A is stimulated, prompting the formation of immature, leaky blood vessels prone to rupture causing hemorrhage and thrombus formation^16,18,20^, which results in plaque instability. The Athero-Express study demonstrated plaque neovascularization and plaque hemorrhage to be significantly related to adverse cardiovascular outcome^21^. Previously, Shah et al.^22^ demonstrated the use of contrast-enhanced ultrasound imaging to detect neovascularization in carotid atherosclerotic plaques and found a moderate correlation with histopathological staining. Practical implication remains a challenge, however, due to lack of an optimal enhancement quantification method and standardized protocols^23^. Accurate detection of the angiogenic process, and thus progression towards a vulnerable plaque, might contribute to the identification of high-risk patients.

Conventional current imaging techniques possess a central role in visualizing the atherosclerotic plaque, such as Computed Tomography Angiography (CT-A) and duplex ultrasound (US) as part of the standard of care^24^. Relatively new in the (clinical) cardiovascular field is near-infrared fluorescence (NIRF) imaging. This modality uses fluorophores as imaging compound instead of the generally used radioactive imaging agents. In and ex vivo near-infrared fluorescent (NIRF) imaging can provide real-time information about molecular tissue composition with high sensitivity^25,26^. In order to image angiogenesis, previously a VEGF-A targeted antibody (bevacizumab) has been conjugated to a NIR fluorescent dye (IRDye800CW) for successful fluorescent imaging in solid tumors (i.e. breast cancer, peritoneal carcinomatosis, esophageal lesions, and colorectal adenomas)^27–30^. Bevacizumab-800CW showed first of all to be safe, to have a high sensitivity and specificity, and allowed for accurate and reproducible imaging of angiogenesis. Previously, our research group has shown that radiolabeled ^89^Zr-bevacizumab is an effective and specific method for the detection of VEGF-A in carotid endarterectomy specimens^31^. The advantage of bevacizumab-800CW over ^89^Zr-bevacizumab is that the former has no radiation burden and is relatively less expensive than the latter. In this study, the feasibility of bevacizumab-800CW-based optical imaging for the detection of carotid plaque instability was assessed. The aim of this study was to determine whether the optical tracer bevacizumab-800CW is suitable for ex vivo visualization of intraplaque angiogenesis by means of identifying VEGF-A presence, and thereby able to distinguish between culprit and non-culprit atherosclerotic plaques.

## Results

### Patient characteristics

Carotid artery plaque specimens from 17 patients were included in the study (*n* = 17), of which three were used for dose-finding analysis. The optimal dose was used in 15 plaques: 11 plaques in the culprit group and three in the non-culprit group, based on the period of time between symptom onset and carotid endarterectomy (CEA), and one plaque was retrieved from an asymptomatic patient and was analyzed separately.

Patient characteristics of the culprit and non-culprit groups are shown in Table 1. Specimens of the common, internal and external carotid artery were used. Of all patients, six suffered from a cerebrovascular accident (CVA), six had a transient ischemic attack (TIA), two suffered from amaurosis fugax, and one patient was asymptomatic but eligible for elective carotid endarterectomy. The time interval between symptom onset and CEA was on average 2 weeks (range: 2–4 weeks) for the culprit group and 7 weeks (range: 5–8 weeks) for the non-culprit group. Some patients did not undergo surgery within the standard of care 2 weeks after symptom onset, caused by either patient or physician delay. The asymptomatic patient was a seventy-year-old male with no cardiovascular risk factors besides uncontrolled hypertension and a BMI of 30.5 kg/m^2^. He had no previous history of cardiovascular problems. Indication for CEA surgery was significant stenosis (≥ 80%) of the carotid artery.

**Table 1:** Patient characteristics and cardiovascular risk factors in culprit and non-culprit group, the asymptomatic patient not included.

Characteristic, *n* (%)	Culprit plaque 11 (77%)	Non-culprit plaque 3 (23%)
**Sex, n (%)**		
Female	3 (27%)	1 (33%)
Male	8 (73%)	2 (67%)
**Age (years)**		
Median (range)	73 (63–79)	67 (66–70)
**Symptomatology, n (%)**		
Cerebrovascular accident	4 (36%)	2 (67%)
Transient ischemic attack	5 (45%)	–
Amaurosis fugax	1 (9%)	1 (33%)
**Time-interval between symptoms and CEA (weeks)**		
Median (range)	2 (2–4)	7 (5–8)
**Degree of stenosis (%)**		
Median (range)	70% (50–99)	80% (70–99)
**Dyslipidemia, n**		
None	–	–
Controlled ≤ 2 drugs	6	3
Uncontrolled or controlled > 2 drugs	5	–
**Diabetes, n**		
None	9	2
Controlled with 1 drug	–	1
Uncontrolled or controlled > 1 drug	2	–
**Hypertension**^**a**^**, *n***		
None	3	1
Controlled ≤ 2 drugs	3	1
Uncontrolled or controlled > 2 drugs	5	1
**BMI (kg/m**^**2**^**)**		
Median (range)	26.2 (20.5–29.4)	26.9 (23.5–33.1)
**Smoking status, n**		
None or last smoked > 5 years	5	2
Current or last smoked ≤ 5 years	6	1
**Cardiovascular history, n**		
None	4	2
Second-degree family	–	–
First-degree family or own history	7	1

CEA, carotid endarterectomy.

^a^Defined as systolic blood pressure ≥ 140 mmHg and/or diastolic blood pressure ≥ 90 mmHg.

### Fluorescent signal assessment

**Figure 1:**
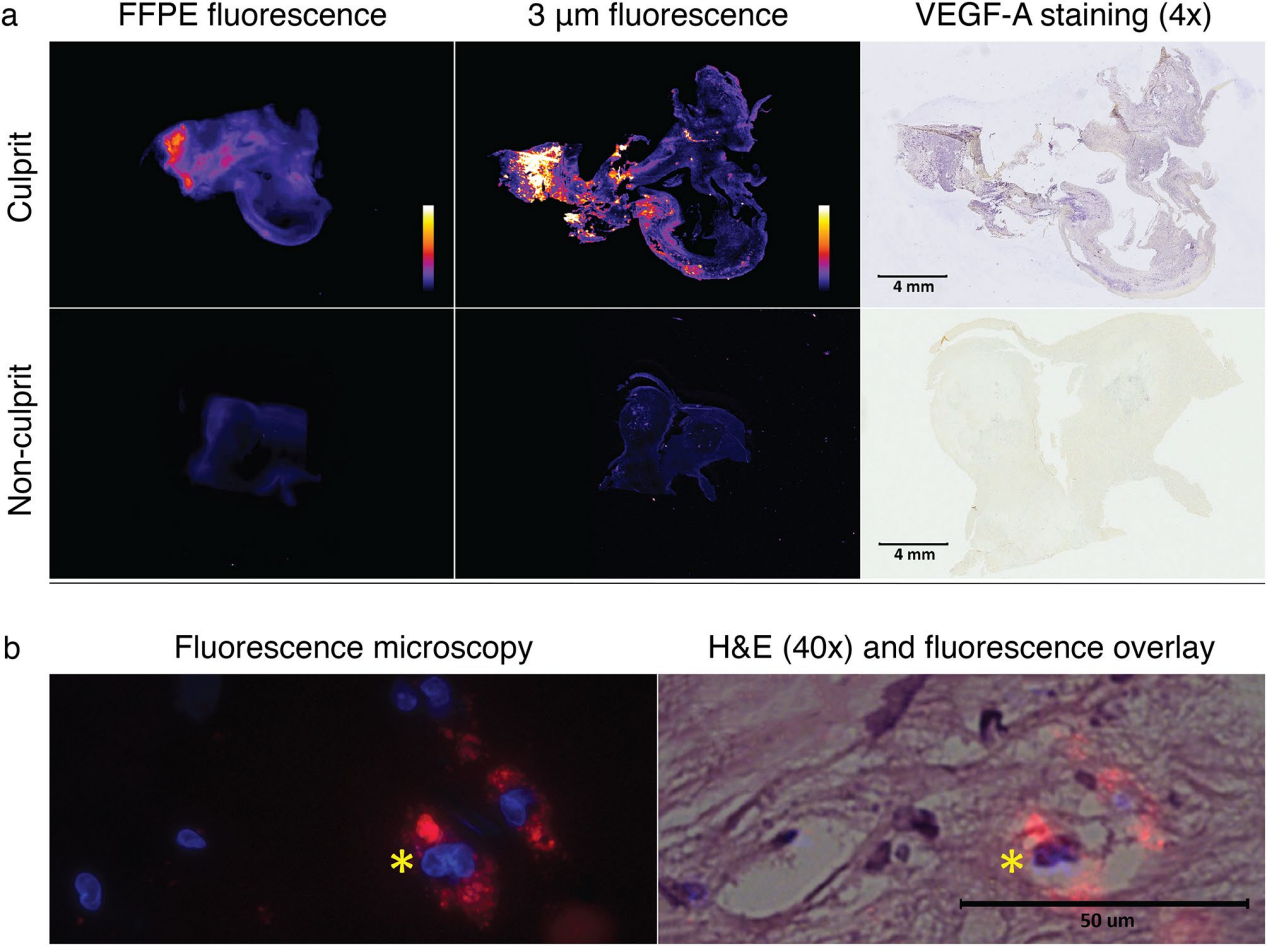
Ex vivo fluorescent signal analyses of a culprit and non-culprit plaque. (**a**) Odyssey (800 nm) scans of a culprit (top row) and non-culprit plaque (bottom row) tissue sections, and the corresponding VEGF-A stainings of the sections (VEGF-A is stained blue). The sections were taken at approximately 1–2 mm depth of the whole tissue specimens. Fluorescent intensity measurements are highest at sites of strong VEGF-A staining, as seen in the culprit plaque. On the contrary, the non-culprit plaque displays low fluorescent intensity and has no visible VEGF-A expression. (**b**) Fluorescence microscopy of a culprit plaque demonstrates the near-infrared signal of the tracer (red; 800 nm channel; left column) overlaps the cell cytoplasm, indicating the intracellular localization of bevacizumab-800CW (H&E staining; right column). Cell nuclei are displayed by Hoechst staining (blue; DAPI channel; left column). The asterisk indicates the same place in the corresponding specimens.

**Figure 2:**
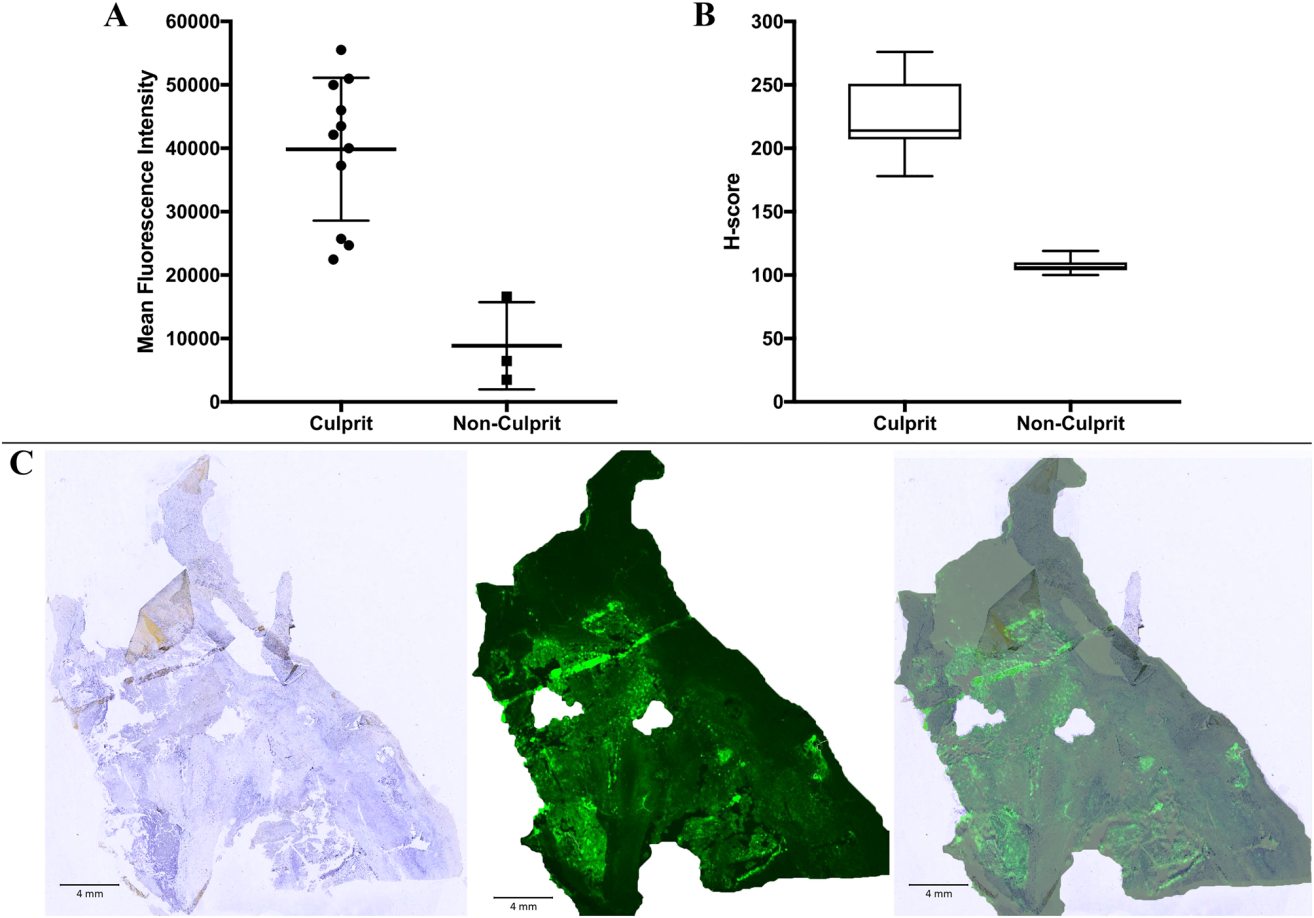
Fluorescent pattern intensity of tissue sections and VEGF-A staining intensity. (**A**) Based on whole tissue fragments, a higher mean fluorescence intensity of all culprit plaques (36,425; range 22,478–55,530) was observed in comparison to non-culprit plaques (8855; range 3515–16,586) after incubation with bevacizumab-800CW. The median MFI of the culprit plaques was higher than the median MFI of the non-culprit plaques (42,129 vs. 6450). (**B**) VEGF-A staining intensities (H-score) of both culprit and non-culprit plaques displayed by a boxplot. The average H-score was the highest in the culprit plaques. (**C**) Representative example of a tissue section taken at approximately 1–2 mm depth of a culprit plaque hot spot displaying the overlap of the pattern of fluorescence and VEGF-A immunohistochemistry (blue staining).

Prior to incubation, specimens were immediately imaged following a standardized imaging protocol to detect potential background signal. PEARL images of background signal in culprit plaques (MFI_background_ = 1961) was similar to non-culprit plaques (MFI_background_ = 1883). Specimens were then incubated within 20 min after surgical excision at room temperature with bevacizumab-800CW 1 µg/ml for 1 h. After incubation, the specimens were carefully rinsed, and subsequently imaged within 10 min. All PEARL images of culprit plaques showed clear fluorescent hot spots (MFI = 36,525; range 22,478–55,530), whereas non-culprit plaques displayed less distinct, weak hot spots (MFI = 8855; range 3515–16,586). MFI was calculated based on three to five measuring points per plaque depending on plaque size. After formalin-fixation and paraffin-embedding (FFPE) and micrometer tissue sectioning, flatbed scanning showed again a clear fluorescence pattern in culprit plaques with clear overlap of VEGF-A expression and the fluorescent signal pattern (Fig. 1a shows a representative example). Based on the H&E staining, fluorescence microscopy revealed the presence of bevacizumab-800CW to be mainly intracellular (Fig. 1b). The corresponding median MFI of the culprit plaques was higher (median MFI = 42,129) than the median MFI of the non-culprit plaques (median MFI = 6450; Fig. 2A), calculated for whole tissue fragments.

### VEGF-A immunohistochemistry

VEGF-A staining was performed in 30 sections of the total number of CEA specimens, with two sections per specimen. The calculated mean H-score of culprit plaques was 223 ± 25.1; the mean of the non-culprit group was 107 ± 6.34. In 91% of the culprit plaques a strong staining intensity (H-score: 201–300) was found. The H-score of the plaque from the asymptomatic patient was 236, indicating an intense staining for VEGF-A. VEGF-A expression levels were observed to be clearly higher in the culprit plaques than in the non-culprit plaques (Fig. 2B). In the culprit plaques, the pattern of fluorescence overlapped with VEGF-A expression in 82% of the specimens (*n* = 9/11) (Fig. 2C).

### Correlation parameters

**Figure 3:**
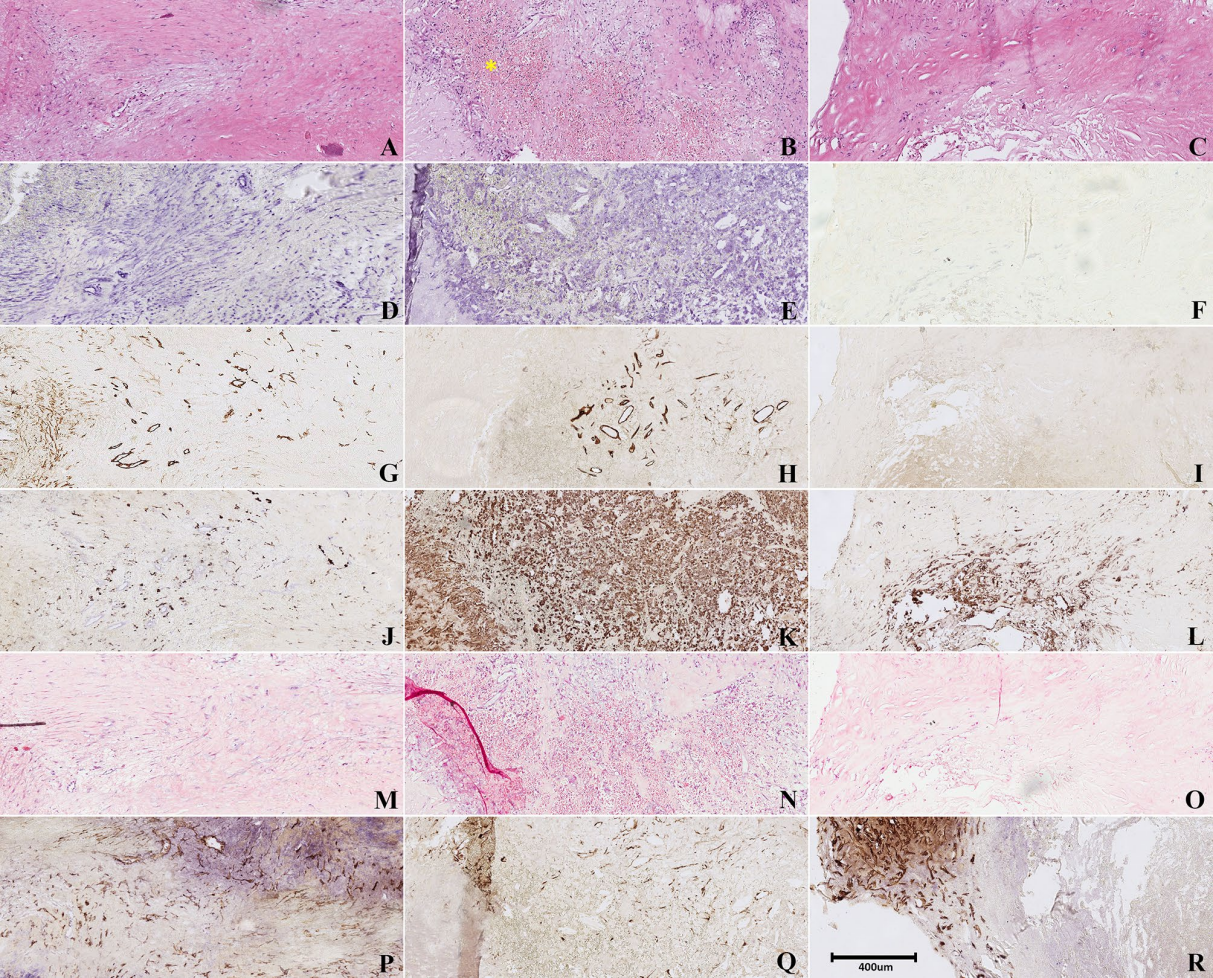
Representative examples of histologic parameters of hot spots of a culprit, the asymptomatic and a non-culprit plaque. The stainings were performed on subsequent slides, depicting a ROI in the specimens. (**A**–**C**) H&E stain, demonstrating extensive intraplaque hemorrhage in the culprit plaque (the yellow asterisks indicate areas with red blood cells, orange spots); (**D**–**F**) VEGF-A stain, revealing elevated VEGF-A expression quantified by the H-score in both the culprit and asymptomatic plaque as opposed to the non-culprit plaque; (**G**–**I**) CD34 stain indicating the association between VEGF-A and microvessel density; (**J**–**L**) CD68 stain, demonstrating an influx of macrophages around the micro vessels in the culprit and asymptomatic plaque; (**M**–**O**) Von Kossa stain, revealing no differences between the culprit, asymptomatic and non-culprit plaques; (**P**–**R**) SMA stain, showing elevated SMA expression in the non-culprit plaque.

Histological examination of the asymptomatic plaque revealed clear pathological features of vulnerability similar to culprit plaques as compared to non-culprit plaques such as intraplaque hemorrhage and intraplaque angiogenesis (Fig. 3). In this particular plaque, intraplaque hemorrhage was present as shown by H&E staining (Fig. 3B), a prominent feature of the vulnerable plaque^15^. The asymptomatic plaque could therefore be deemed vulnerable, i.e. culprit, as these features are characteristic for culprit lesions^32^. In Table 2, an overview of the various associations between VEGF-A staining and correlation parameters is displayed.

**Table 2:** correlation parameters for VEGF-A staining and CD34, CD68, SMA and Von Kossa staining.

Characteristic	Culprit specimens, *n* (%)	Non-culprit specimens, *n* (%)	Chi square tests of independence^a^
CD34 stain	15/15 (100%)	0/6	χ^2^ (1) = 10.313 *p* = 0.001 ϕ = 0.829 *n* = 21
CD68 stain	15/17 (88%)	1/4 (25%)	χ^2^ (4) = 11.030 *p* = 0.026 ϕ = 0.606 *n* = 21
SMA stain^b^	2/17 (11%)	6/6 (100%)	χ^2^ (4) = 11.341 *p* = 0.023 ϕ = 0.870 *n* = 23
Von Kossa stain	16/22 (72%)	3/6 (50%)	χ^2^ (1) = 1.116 *p* = 0.291 ϕ = 0.200 *n* = 28

^a^The Chi Square tests of independence were calculated based on the VEGF-A ROIs found in the specimens and the intensity of the various stainings in those ROIs. The asymptomatic plaque was not taken into account.

^b^For the association between SMA and VEGF-A staining, an inverse Chi Square test was used.

CD34 staining for microvessel density confirmed the presence of microvasculature in all culprit plaques. In addition, in all culprit plaques, including the asymptomatic plaque, VEGF-A staining was strongest in areas of high microvessel density. A strong association with VEGF-A staining was demonstrated (ϕ = 0.829; *p* < 0.001). No intraplaque micro vessels were observed in the non-culprit plaques.

Von Kossa staining for (micro)calcifications was performed in 22 sections of the culprit plaques (2 per specimen), and revealed small calcium deposits around the VEGF-A and micro vessel areas in 73% of the sections (*n* = 16/22). Of the non-culprit plaques, 6 sections were stained with Von Kossa and 3 specimens revealed microcalcifications in both the intimal and medial layers. No significant association was found between microcalcifications and VEGF-A expression in both the culprit and non-culprit group (*p* > 0.05).

Macrophage infiltration (CD68 staining) was most abundant in the VEGF-A ROIs in culprit plaques (*n* = 15/17). Weak to moderate staining was found in non-culprit plaques. A moderate association was found between VEGF-A expression scores and CD68 staining scores (ϕ = 0.606; *p* < 0.05). The average CD68 score in the culprit group was higher than in the non-culprit group (2.83 ± 0.389 vs 1.27 ± 0.577).

Strong expression of smooth muscle actin (SMA) staining was observed in the non-culprit plaques as opposed to mostly weak staining in culprit plaques. The asymptomatic plaque also displayed low SMA expression. There was a strong inverse correlation between H-scores and SMA staining (ϕ = 0.870; *p* = 0.023).

## Discussion

Optical imaging is a relatively new modality in the clinical cardiovascular field and can be used for real-time detection of pathological processes^13,31,33^. Currently, no decisive method is available to make a distinction between culprit and non-culprit atherosclerotic plaques. This study investigated the feasibility of using bevacizumab-800CW for the identification of vulnerable plaques by determination of their VEGF content. We demonstrated a successful method to detect a measurable fluorescent signal in incubated atherosclerotic plaques. Next, the optimal incubation concentration was determined, 1 µg/ml bevacizumab-800CW. We were able to demonstrate that areas of high VEGF-A expression, based on specific immunohistochemistry, correlated with a high fluorescence signal. Strong VEGF-A expression was observed in culprit plaques, in contrast to the weak staining found in non-culprit plaques.

We demonstrated the relationships between VEGF-A expression, and macrophage infiltration and intraplaque microvessel density. Although macrophage infiltration by itself does not appear to be predictive of upcoming acute events in the future^15^, we found that it coincides with VEGF-A expression and intraplaque angiogenesis. No correlation was demonstrated between microcalcifications and VEGF-A expression. Furthermore, microcalcifications were present both in culprit and non-culprit plaques. This corresponds to previous research by Hop et al.^13^, who investigated the use of the PET-tracer ^18^F-NaF specific for microcalcifications in culprit and non-culprit carotid plaques ex vivo.

The inverse association found between VEGF-A expression and SMA expression demonstrates that SMA expression is more pronounced in non-culprit plaques, indicating these plaques to contain more mature vascular smooth muscle cells (VSMCs). This complements earlier research in which diminished SMA staining was related to unstable coronary plaques^34^. Additionally, Shinde et al.^35^ demonstrated that SMA-expressing myofibroblasts are associated with scar contraction in healing myocardial infarcts. A possible explanation why SMA expression in particular is present in non-culprit plaques is that these myofibroblasts take part in wound healing processes and vessel repair, that presumably stabilize the atherosclerotic plaque^36^. Although VSMCs and myofibroblasts are distinct cell types, SMA may have protective properties in the sense that SMA expression stabilizes the atherosclerotic plaque.

The fluorescent signals found at sites of clear visual macrocalcification without VEGF-A expression at those specific sites could be explained by autofluorescence of calcified areas, as described previously in human atherosclerotic arteries^37^. Conversely, the frail integrity of 3 µm tissue slides results in disrupted tissue sections due to tearing and breaking of the tissue. This could attribute for a weaker bevacizumab-800CW fluorescent signal, imaged by the flatbed scanner, at the corresponding sites of VEGF-A expression. Another reason could be the submersion method of incubation with bevacizumab-800CW as the tracer might not be able to penetrate the full thickness of the specimens.

A limitation of this study is the low number of asymptomatic and non-culprit atherosclerotic plaques, which posed a difficulty in comparing symptomatic to non-symptomatic plaques and assessing statistical significance between culprit and non-culprit plaques. Asymptomatic plaques are normally not resected as standard of care because of the patient risk involved in the procedure. The number-needed-to-treat (NNT) to prevent major CVA or death is 6 for symptomatic patients, whereas asymptomatic patients have an NNT of 20, emphasizing patient risk^38,39^. Additionally, symptomatic plaques are normally removed within 2 weeks after symptom onset as standard of patient care in the Netherlands, which could be an explanation for the low number of non-culprit plaques.

This study complements previous research by our group with ^89^Zr-bevacizumab and the use of bevacizumab in CEA specimens^31^, as the monoclonal antibody bevacizumab was able to target VEGF-A ex vivo in human carotid atherosclerotic plaques in this study too. In addition, this study adds knowledge as we demonstrate the ability of the conjugate bevacizumab-800CW to identify intraplaque angiogenesis by targeting VEGF-A, and to be capable of distinguishing between culprit and non-culprit plaques. Furthermore, we demonstrate that angiogenesis correlates with macrophage infiltration using CD68 immunohistochemistry. An advantage is that a systemic dose of bevacizumab-800CW is already proven to be safe and effective in humans, according to various cancer studies^27–30^, which facilitates further in vivo research in the cardiovascular field. In general, the benefit of NIRF-tracers over PET-tracers is that the production process of NIRF-tracers is generally simpler and less expensive, due to the long shelf life and the elimination of radioactive infrastructure in the production process. NIRF-imaging allows for non-invasive imaging techniques, which is favorable in this fragile patient group. Additionally, with NIRF imaging, it is easier to image in real-time, there is no radiation burden, and the imaging time itself is relatively short^31^, however whole body imaging and deep signal penetration is lacking.

In conclusion, the NIRF tracer bevacizumab-800CW is promising in visualizing VEGF-A expression in human carotid atherosclerotic plaques ex vivo. Our results demonstrate the potential of bevacizumab-800CW to define vulnerable (VEGF-positive) and non-vulnerable (VEGF-negative) plaques. The validation of the latter hypothesis should take place in a clinical study. Future studies should focus on the clinical proof of principle in vivo of the use of bevacizumab-800CW in this particular patient group, to allow for its validation in vivo*.* We believe that this tracer might allow for improved risk-stratification of carotid atherosclerosis and therefore a reliable selection method for CEA. Benefits would be prevention of disease and mortality caused by acute vascular events. Ultimately, clinical implementation of this tracer to select for CEA should rely on the predictive value of angiogenesis in high-risk plaques.

## Methods

### Study design

For this ex vivo feasibility study, fresh human carotid specimens were obtained from patients undergoing carotid endarterectomy between September 2017 and April 2018 in the University Medical Center Groningen (UMCG). Previous literature demonstrated that ‘positive’ plaques corresponded to the patients with recent events (i.e. culprit plaques) and ‘negative’ plaques to patients with a remote history of ischemic stroke (i.e. non-culprit plaques)^32,40^. Odds ratios of present plaque characteristics as risk indicators for stroke were demonstrated to be significantly greater for plaques retrieved from patients operated within 30 days after the ischemic event than in plaques retrieved after 30 days after symptom onset. For high microvessel density, these odds ratios were 4.52 and 1.47, respectively^32^. In this study, we adopted this dichotomy: ‘positive’ culprit plaques were defined as plaques in patients with recent events (i.e. cerebrovascular accident, transient ischemic attack, amaurosis fugax), and ‘negative’ non-culprit plaques were defined as plaques in patients with no events in the last 4 weeks. For all patients, clinical and demographic data were collected from medical records, using the hospital’s electronic medical record system, including medication use and cardiovascular risk factors that were present before symptom onset. The study was released by the Medical Ethical Committee of the UMCG and is in accordance with the Helsinki Declaration (2013). It was decided that patient informed consent was not required as the study uses waste tissue-material, that is not restricted by the Dutch WMO (Wet Mensgebonden Onderzoek, which translates to ‘law on research involving human subjects). The IRB of the UMCG approved this study. For all patients, it was checked if objection statements about the use of waste material were ever recorded by the hospital. Data analysis was performed anonymously.

Primary endpoints were the accumulation of ex vivo incubated bevacizumab-800CW in carotid plaques by fluorescence macro- and microscopy, and its correlation with VEGF-A expression, macrophage infiltration (CD68), microvessel density (CD34), microcalcifications (Von Kossa) and structural integrity of the vessel wall (smooth muscle actin; SMA), determined by semi-quantitative analysis of (immuno)histochemical stainings. For these parameters, culprit and non-culprit plaques were compared.

### Fluorescence imaging techniques

Bevacizumab (Avastin; Genentech, South San Francisco CA, USA) conjugation with the fluorescent dye IRDye800CW was performed at the department of Clinical Pharmacy and Pharmacology, as described earlier^41^. An optimal dose of 1 μg/ml was determined by submersing the specimens in various solutions (4 μg/ml, 2 μg/ml, and 1 μg/ml bevacizumab-800CW in PBS) for one hour in a dark room to prevent photobleaching. The used concentrations were based on theoretical blood concentrations after intravenous administration of 4.5 mg, 10 mg, and 25 mg bevacizumab-800CW, respectively. The imaging procedures with the optimal dose were as follows. Immediately after excision, the specimens were rinsed with PBS. Subsequently, images of the whole tissue were acquired with the PEARL imager (LI-COR Biosciences, Lincoln NE, USA). The specimens were then submersed in a 1 μg/ml bevacizumab-800CW solution for one hour at room temperature. PEARL images were obtained from the whole tissue specimen, after which hot spots (i.e. spots with 95% of the highest fluorescent signal (expressed in Mean Fluorescence Intensity [MFI]) were selected. The MFI was only calculated from the subsequent PEARL images. All images of all specimens were scaled to the same intensity. The spots were formalin-fixed, paraffin-embedded (FFPE) and cut in 3 μm sections.

The FFPE blocks were analyzed with the Odyssey fluorescent flatbed scanner (LI-COR Biosciences, Lincoln NE, USA). In order to cross-correlate a fluorescent signal within the corresponding 3 μm slides with additional histopathological staining, the sections were deparaffinized (10 min xylene) and analyzed with the Odyssey scanner. Afterwards, these 3 μm slides were stained with hematoxylin and eosin (H&E) for cell morphology and to determine what part of the plaque displayed fluorescent signal.

In additional consecutive 3 μm slides cell nuclei were visualized with Hoechst staining (Invitrogen, Carlsbad CA, USA) in the same manner as we described previously^29^. Leica NIR fluorescence microscopy was carried out to colocalize bevacizumab-800CW and VEGF-A. For image processing and analysis, LAS-AF software (Leica Microsystems, Wetzlar, Germany) and Fiji/ImageJ (version 1.8.0., LOCI, University of Wisconsin, USA) were used as described previously^42^.

### (Immuno)histochemistry

To allow for examination of the pathological status (i.e. vulnerability) of the plaques, for each spot slides were double stained for VEGF-A and either Von Kossa, CD34, CD68 or SMA. The latter three immunohistochemical stainings were performed by standardized machine protocols. For the staining of microvessels, slides were incubated with anti-CD34 (mouse IgG_1_ κ monoclonal, Dako, clone QBEnd/10). Slides were incubated with anti-CD68 (mouse IgG_3_ κ monoclonal, Dako, clone PG-M1) for the staining of macrophages. Smooth muscle actin in the vessel walls was stained with anti-SMA (mouse monoclonal, Dako, clone 1A4). All slides were incubated for one hour at room temperature. Consecutively, the slides were incubated with horseradish peroxide (HRP) secondary antibodies, and afterwards chromogenic staining with 3,3′-diaminobenzidine (DAB) was performed for color development. Hematoxylin was used to counterstain the slides.

For VEGF-A, slides were deparaffinized and rehydrated with xylene (10 min) and graded alcohols. For heat-induced antigen retrieval (15 min), 100 mM Tris/EDTA buffer (pH 9.0) was used. Endogenous peroxidase was blocked for 30 min with 30% hydrogen peroxide, followed by avidin–biotin blocking. Subsequently, the slides were incubated overnight at 4 °C with a polyclonal rabbit anti-human VEGF-A antibody (RB9031, Thermo Fisher Scientific, Waltham, MA, USA). Slides were incubated with goat anti-rabbit biotin in phosphate-buffered saline with 1% bovine serum albumin [PBS 1%BSA] with 1% AB serum, and consecutively with alkaline-phosphatase conjugated streptavidin in PBS 1% BSA with 1% AB serum, both for 30 min. For color development, alkaline-phosphatase activity was visualized using naphthol AS-MX phosphate and Fast Blue salt (30 min). The slides were washed with demineralized water and cover slipped with Kaiser’s glycerin. PBS was used for washing, and all steps were performed at room temperature unless indicated otherwise.

For the Von Kossa staining, the slides were first submersed with 1% silver nitrate solution for 60 min in broad daylight, followed by 3% sodium thiosulfate (5 min). The slides were counterstained with nuclear fast red (3 min), after which dehydration was performed with graded alcohols. The slides were then air-dried (30 min) and cover slipped with mounting medium. Demineralized water was used throughout washing.

The stained slides were digitized with the NanoZoomer Digital Pathology Scanner (Hamamatsu Photonics K.K., Hamamatsu City, Japan). The imaged slides were then analyzed and evaluated with Aperio ePathology (Leica Biosystems, Wetzlar, Germany) software. To score the degree of VEGF staining, the H-score was calculated in the same manner as described previously by our group^30^, determined by semi-quantitative combined assessment of the percentage of stained cells and the staining intensity (0–300; continuous scale; 0–100: negative/weak; 101–200: moderate; 201–300: strong). Von Kossa, CD34, CD68 and SMA stainings were categorized semi-quantitatively (score 1 = weak; 2 = moderate; 3 = strong). The pattern of fluorescent signal was compared to corresponding VEGF-A-stained slides by fitting the VEGF-A region of interest (ROI) on the corresponding fluorescence image and determining the extent of overlap between VEGF-A expression and fluorescent signal.

### Data and statistical analysis

Data were analyzed with SPSS Statistics 24 (IBM, Armonk, NY, USA) and Fiji/ImageJ (version 1.8.0., LOCI, University of Wisconsin, USA). Regions of interest (ROIs) were drawn based on the VEGF-A staining (95% of the strongest expression, determined by standardized counting algorithms). The mean fluorescence intensity of this ROI was measured quantitatively. Categorical data was tested with Chi-Square association tests. A *p *value ≤ 0.05 was considered to be statistically significant.

## Abbreviations

BSA: Bovine serum albumin
CEA: Carotid endarterectomy
CT-A: Computed tomography angiography
CVA: Cerebrovascular accident
DAB: 3,3′-Diaminobenzidine
FFPE: Formalin-fixed paraffin-embedded
HRP: Horseradish peroxidase
H-score: Continuous scale by combining staining intensity and percentage of stained cells
H&: Hematoxylin and eosin
IHC: Immunohistochemistry
MFI: Mean fluorescence intensity
NIRF: Near-infrared fluorescence
PBS: Phosphate-buffered saline
ROI: Region of interest
SMA: Smooth muscle actin
TIA: Transient ischemic attack
US: Ultrasound
VEGF-A: Vascular endothelial growth factor A
